# Dentigerous cyst: enucleation or marsupialization? (a case report)

**DOI:** 10.11604/pamj.2021.40.149.28645

**Published:** 2021-11-10

**Authors:** El Gaouzi Rajae, El Harti Karima

**Affiliations:** 1 Department of Oral Surgery, Ibn Sina Treatment and Consultation Center, Faculty of Dentistry, Mohammed V University, Rabat, Morocco

**Keywords:** Dentigerous cyst, enucleation, marsupialization, case report

## Abstract

Dentigerous cysts, also called follicular cysts, are slow-growing benign odontogenic cysts that are thought to be developmental in origin. On imaging, they usually present as a well-defined and unilocular radiolucency surrounding the crown of an unerupted or impacted tooth. This article presents a case of unilateral mandibular dentigerous cysts associated with unerupted mandibular canine in a healthy patient treated by enucleation, along with a review of the literature and an examination of the treatment modality. The aim of this paper is to highlight how to choose the adequate treatment for dentigerous cyst cases.

## Introduction

Dentigerous cysts, also known as follicular cysts, are the second most common form of benign developmental odontogenic cysts that results from accumulation of fluid between reduced enamel epithelium and the crown of an unerupted tooth. Dentigerous cysts occur over a wide age range with a peak frequency in the second to fourth decades, and they are the second most common odontogenic cysts after radicular cysts, accounting for approximately 24% of all true cysts in the jaws [1]. They are uncommonly seen in childhood because they almost exclusively occur in secondary dentition. The most frequently involved tooth is the mandibular third molar followed by the maxillary canine, and mandibular premolars. Dentigerous cysts associated with unerupted mandibular canine are less common.

The dentigerous cysts progress slowly, they are usually asymptomatic and discovered incidentally during a routine radiographic examination, however, they may be large and result in a palpable mass. Additionally, as they grow, they displace adjacent teeth. The most common clinical complication is paresthesia of the inferior alveolar nerve.

Dentigerous cysts can be treated by enucleation or marsupialisation. The treatment decision takes into account different criteria, including cyst size, cyst location, removal of unerupted tooth, and follow-up possibilities. Usually, large dentigerous cysts are treated by marsupialisation. The aim of the present study is, to present a clinical case of a large dentigerous cyst associated with unerupted mandibular canine in young female treated by enucleation, and to highlight the treatment making-decision.

## Patient and observation

**Patient information:** a systematically healthy 25-year-old male patient presented for consultation to the Department of Oral Surgery of Ibn Sina Dental Consultation and Treatment Center in Rabat, Morocco. The patient's chief complaint was a swelling over the anterior sector of his mandible, accompanied by a discomfort sensation, which first appeared a month before the consultation. The patient did not mention any toxic (deleterious) habits, and reported no contributory significant dental history.

**Figure 1:**
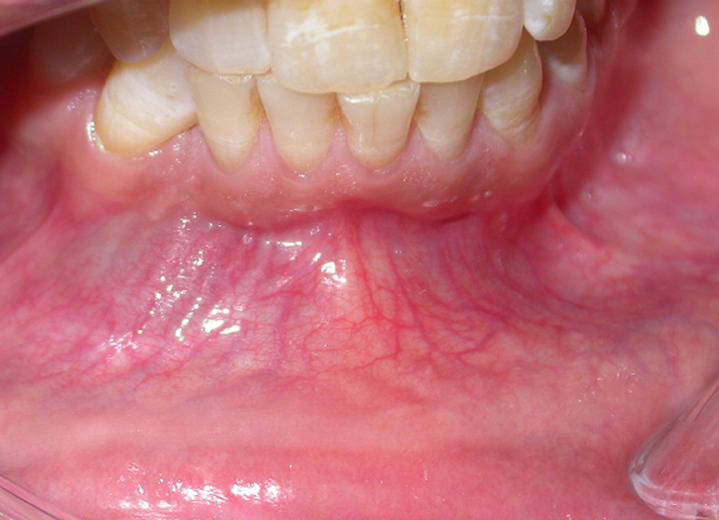
preoperative intraoral view showing a swelling in the anterior region of the mandible

**Figure 2:**
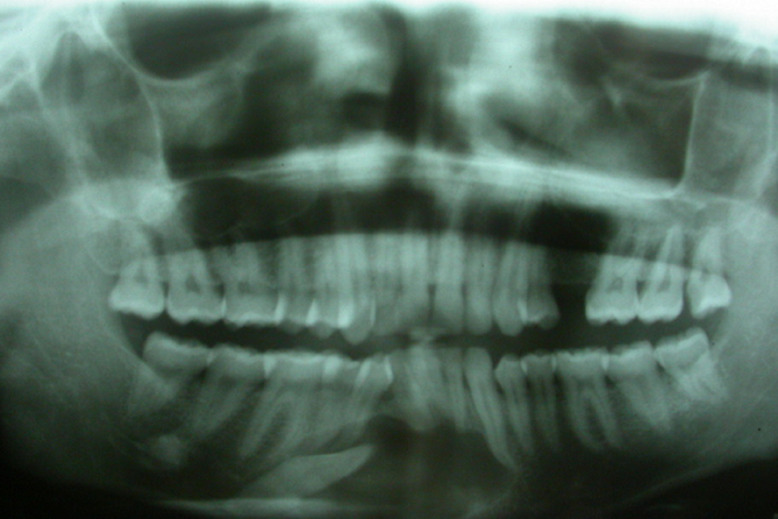
preoperative orthopantomography showing an oval radiolucent lesion in mandible

**Clinical findings:** local examination appeared to be normal, but intra-oral one (Figure 1), revealed a palpable vestibular swelling expanded from the mandibular right canine region to the mandibular left canine region. On palpation, the swelling was painless, firm, and the overlying mucosa did not show any inflammatory signs. All permanent teeth were present except for the mandibular right canine. Lymph node examination ruled out the presence of any pathology. Orthopantomograph (OPG) (Figure 2) revealed a well-defined oval radiolucent lesion, extending from the mesial of mandibular right first molar, to the left canine, upon 1 cm from the inferior border of the mandible inferiorly. Radiolucency included the right permanent canine.

**Diagnostic assessment:** clinical differential diagnosis included periapical cyst, keratocystic odontogenic cyst (KOT) and ameloblastoma. As there was no carious lesion seen clinically, periapical cyst was ruled out. Dentigerous cyst was the first choice of diagnosis as the radiograph revealed unilocular radiolucency surrounding the neck of the crown of an unerupted tooth, with diffuse and thin corticated borders, which are the radiographical features usually seen in dentigerous cyst.

**Figure 3:**
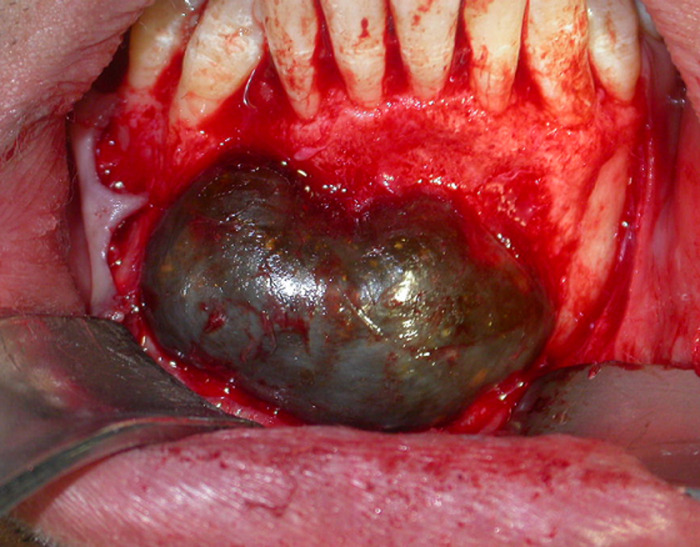
intra oral exposure of cystic lesion

**Figure 4:**
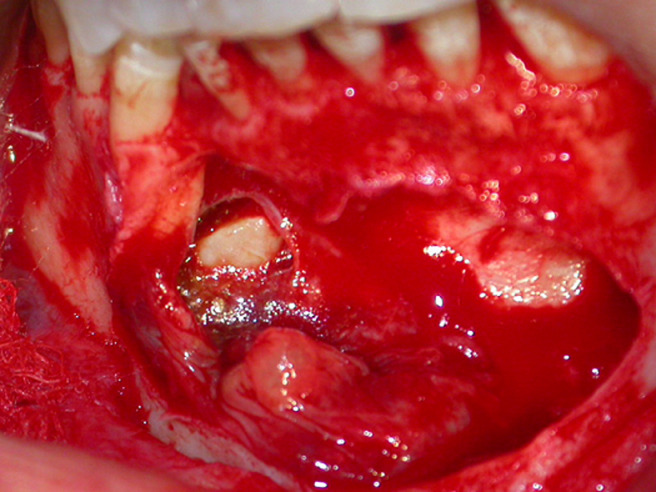
view of the right mandibular permanent canine after enucleation of the cyst

**Figure 5:**
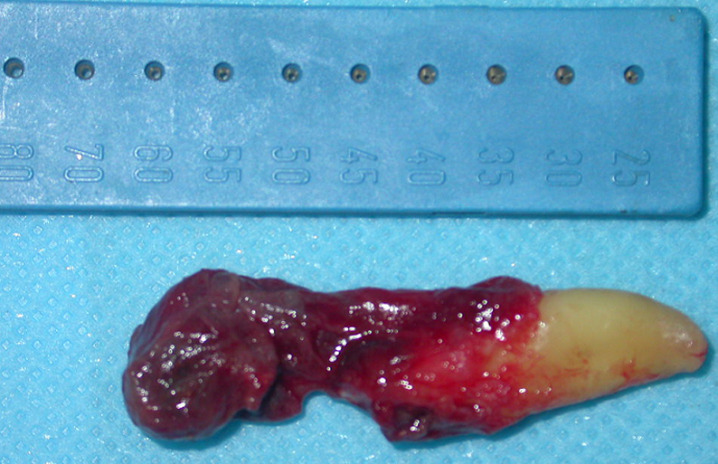
enucleated specimen

**Therapeutic intervention:** under local anesthesia with articaine 68 mg/1.7 ml with 1/200,000 adrenaline, horizontal incision and double releasing incisions were done. Then, the mucoperiosteal flap was reflected. The soft cystic tissue was exposed (Figure 3), and a complete enucleating of the cysts was performed with removal of the permanent canine (Figure 4). Incision was closed using 3-0 silk suture, and the specimen was sent for histopathological investigation (Figure 5).

**Follow up and outcomes:** histopathological report revealed a dentigerous cyst. The patient was seen after one week to remove sutures, and to control health process.

**Informed consent:** written informed consent was obtained from the patient to publish anonymized information in this article.

## Discussion

Dentigerous cysts (DCs) have been reported extensively in the literature. The exact cause of this cyst is still unknown, but many theories are proposed. The “intrafollicular theory” suggests that a DC is a consequence of fluid accumulation between the outer and inner surfaces of the epithelium. This accumulation occurs during the formation of the crown. The second theory is the “enamel hypoplasia theory”. It suggests the development of the cyst after stellate reticulum degeneration. “Main´s theory” suggests that the cyst is a result of the hydrostatic pressure exerted by an impacted tooth on the follicle which results in the separation of the impacted crown from the surrounding follicle [2]. A dentigerous cyst can be inflammatory or noninflammatory. The inflammatory type of dentigerous cyst occurs because of inflammation in a nonvital deciduous tooth. The noninflammatory type develops due to the pressure exerted by the erupting tooth on an impacted follicle [3].

Radiographically, dentigerous cysts are suspected when the size of the follicular space is larger than 5 mm. Radiographically, dentigerous cyst presents as well-defined unilocular radiolucency, often with a sclerotic border and this radiolucency surrounds the crown of an impacted tooth [4].

These cysts are commonly single lesions. Bilateral and multiple dentigerous cysts are very rare although they have been reported in patients with syndromes such as basal cell nevus syndrome, mucopolysaccharidosis, and cleidocranial [5]. The differential diagnosis may include odontogenic keratocysts, primordial cysts, and odontogenic tumors (pindborg tumor, adenomatoid odontogenic tumor, mural ameloblastoma, unilocular ameloblastoma, ameloblastic fibroma, odontomas, and cementomas) [6]. In its histopathology, DC epithelium consists of 2-4 layers of smooth nonkeratinized cells and the interface of epithelium and connective tissue is smooth. There may be mucous, ciliated columnar and fat cells in DC epithelium [7].

Several treatment options include marsupialization and complete enucleation. Marsupialization is the conversion of a cyst into a pouch by suturing the cyst lining to the oral mucosa. This conservative method is used, if the preservation of the displaced teeth is desirable, especially in a young patient. It is also used if the cyst is large and there is a possibility of destruction of the surrounding tissue and a pathologic fracture of the mandible [8,9].

This method has fewer complications than enucleation regarding the preservation of important anatomical structures and developing permanent tooth germs. The disadvantage of marsupialization is the pathologic tissue left in situ. Ameloblastoma, squamous cell carcinoma, or intraosseous mucoepidermoid carcinoma may develop from the cells in the lining of a dentigerous cyst; however, recurrence of dentigerous cyst is seldom found, especially after complete removal of cyst or tooth eruption [9]. Enucleation is a radical method for removing all the cystic capsule. The chosen treatment plan whenever the cyst is small, and saving the involved tooth is impossible [10].

In our case, even if the cyst was large, enucleation was the most widely accepted procedure. Because, the patient had a low socio-economic situation and she will have to travel for a long time, making follow-up visits impossible. Besides, there was no risk of fracture of the jaw.

Many authors differ in their opinion with regard to enucleation of large dentigerous cysts. This is largely due to the fact that larger cystic cavities lack organization of a blood clot and formation of new bone is questionable. A blood clot in a devitalized area is a great risk, as it can easily become infected and may lead to the unwanted consequences of local inflammation [1].

However, various studies have shown predictable spontaneous bone regeneration in young patients after enucleation of such large cysts [1,11,12]. Many authors believe that bone grafting in young patients should be considered carefully and in most of the instances it is unnecessary [1].

## Conclusion

From our experience, large dentigerous cysts can be treated by enucleation. Therefore, the treatment decision must be taken appropriately for each case taking into account anatomic site, clinical extent, size, age, and follow-up possibility.

## References

[1] Arakeri G, Rai KK, Shivakumar HR, Khaji SI (2015). A massive dentigerous cyst of the mandible in a young patient: a case report. Plast Aesthet Res.

[2] AlKhudair B, AlKhatib A, AlAzzeh G, AlMomen A (2019). Bilateral dentigerous cysts and ectopic teeth in the maxillary sinuses: a case report and literature review. Int J Surg Case Rep.

[3] Zerrin E, Husniye DK, Peruze C (2014). Dentigerous cysts of the jaws: clinical and radiological findings of 18 cases. J Oral Maxillofac Radiol.

[4] Vasiapphan H, Christopher PJ, Kengasubbiah S, Shenoy V, Kumar S, Paranthaman A (2018). Bilateral dentigerous cyst in impacted mandibular third molars: a case report. Cureus.

[5] Ghandour L, Bahmad HF, Bou-Assi S (2018). Conservative treatment of dentigerous cyst by marsupialization in a young female patient: a case report and review of the literature. Case Rep Dent.

[6] Duhan R, Tandon S, Vasudeva S, Sharma M (2015). Dentigerous cyst in maxillary sinus region: a rare case report and outline of clinical management for paediatric dentists. IOSR J Dent Med Sci.

[7] Jain N, Gaur G, Chaturvedy V, Verma A (2018). Dentigerous cyst associated with impacted maxillary premolar: a rare site occurrence and a rare coincidence. Int J Clin Pediatr Dent.

[8] Khandeparker RV, Khandeparker PV, Virginkar A, Savant K (2018). Bilateral maxillary dentigerous cysts in a nonsyndromic child: a rare presentation and review of the literature. Case Rep Dent.

[9] Hu YH, Chang YL, Tsai A (2011). Conservative treatment of dentigerous cyst associated with primary teeth. Oral Surg Oral Med Oral Pathol Oral Radiol Endod.

[10] Berden J, Koch G, Ullbro C (2010). Case series: treatment of large dentigerous cysts in children. Eur Arch Paediatr Dent.

[11] Bonardi JP, Gomes-Ferreira PHS, De Freitas Silva L, Momesso GAC, De Oliveira Sabrina Ferreira D, Dos Santos Pereira R (2017). Large dentigerous cyst associated to maxillary canine. J Craniofac Surg.

[12] Önay Ö, Süslü AE, Yilmaz T (2019). Huge dentigerous cysts in the maxillary sinus: a rare case in childhood. Turk Arch Otorhinolaryngol.

